# Insight into the Mechanism of Cobalt-Nickel Separation Using DFT Calculations on Ethylenediamine-Modified Silica Gel

**DOI:** 10.3390/ma16093445

**Published:** 2023-04-28

**Authors:** Hailun Yang, Ling Yuan, Menglei Yuan, Pengge Ning

**Affiliations:** 1 SINOPEC Research Institute of Petroleum Processing Co., Ltd., Beijing 100083, China; yanghailun.ripp@sinopec.com; 2 National Key Laboratory of Biochemical Engineering, CAS Key Laboratory of Green Process and Engineering, Beijing Engineering Research Center of Process Pollution Control, Institute of Process Engineering, Innovation Academy for Green Manufacture, Chinese Academy of Sciences, Beijing 100190, China; 3 State Key Laboratory of Pollution Control and Resource Reuse, School of the Environment, Nanjing University, Nanjing 210023, China; 4 State Key Laboratory of Solidification Processing, School of Materials Science and Engineering, Northwestern Polytechnical University, Xi’an 710072, China

**Keywords:** adsorptive separation, ethylenediamine, silica gel, DFT calculation, separation mechanism

## Abstract

The separation of Co(II) and Ni(II) from leaching solution is gaining interest because Co(II) and Ni(II) are increasingly used in emerging strategic areas, such as power batteries. Herein, the surface of silica gel is functionalized with 1,2-ethylenediamine and used for the separation of Co(II) and Ni(II). The Co(II) removal efficiency of the modified silica is 80.2%, with a 4-fold improvement in the separation factor. The geometry, frequency, and electrostatic potential of the ethylenediamine modified silica gel (en/SG) are calculated. The corresponding properties of M^2+^ (M-Co, Ni) adsorbed on en/SG in an aqueous solution are simulated and analyzed. The results show that ethylenediamine tends to form [Men(H_2_O)_4_]^2+^ after binding to M^2+^, and the binding ability of Co(II) to ethylenediamine is stronger. Besides, the thermodynamic calculations show that en/SG has a more negative Gibbs free energy when absorbing Co(II) in aqueous solution, so en/SG is more inclined to bind with Co(II) preferentially. It is the difference in complexation ability between Ni, Co, and ethylenediamine that enlarges the difference in the original physical adsorption, thus strengthening the separation performance. This work will provide guidance for a rational design of high-performance nickel-cobalt adsorption materials.

## 1. Introduction

With the growth of the electric vehicle rechargeable battery market, the consumption of cobalt and nickel is expected to increase dramatically, which may lead to a significant increase in their supply risks [1,2,3,4]. Therefore, improving the recovery efficiency of cobalt and nickel in secondary resources is one of the sustainable strategies to alleviate its supply risk [5,6,7,8]. Generally, cobalt and nickel coexist (e.g., spent lithium-ion batteries) and are transferred into aqueous solution via leaching using reagents such as H_2_SO_4_ [9,10]. Extraction and adsorption are two common techniques for separating nickel and cobalt [11,12,13]. Since the adsorption technology does not require saponification in the separation process of nickel and cobalt, it avoids the generation of salty wastewater and shows potential advantages [14,15,16]. Adsorption materials are divided into organic materials and inorganic materials, such as chelating resin [17], cellulose [18], chitosan [19], inorganic silicone [20], activated carbon [21], etc. Among them, inorganic silica gel has been widely studied due to its excellent characteristics, such as high mechanical strength and stable physicochemical properties [22,23]. Although its specific surface area is relatively large, the inorganic silica gel without surface modification has low capacity and selectivity for metal ions [24,25,26]. Therefore, researchers have tried to modify inorganic silica gel, which combines the advantages of inorganic silica gel and organic groups [27,28]. In recent years, a variety of silica hybrid materials with a high adsorption capacity for heavy-metal ions have been studied, including the ethylenediamine silica hybrid, thiourea silica hybrid, polyallylamine silica hybrid, etc [29].

Ethylenediamine (en), as the simplest aliphatic diamine, is widely used in pesticides, pharmaceuticals, dyes, rubber, and so on [30,31]. Because the N atom containing lone pair electrons in ethylenediamine can coordinate with metal ions and crosslink with other groups, it is often used for surface modification of solid carriers [32,33]. For example, aminopropyltrimethoxy silane, methyl acrylate, and ethylenediamine were used to prepare polyamide-amine dendrimers on the surface of inorganic silica and their sorption properties for Ag^+^ were investigated [34]. Four aliphatic polyamines, including ethylenediamine, were used to modify the surface of silica and the sorption properties of modified silica for Pb^2+^, Co^2+^, and Ni^2+^, which were investigated [35]. Arakaki et al. produced ethylenediamine modified silica by grafting ethylenediamine onto inorganic silica using choropropyltrimethoxysilane. The sorption properties of Cu^2+^, Co^2+^, and Ni^2+^ and the thermodynamic data of the sorption process for ethylenediamine modified silica were investigated [36]. In addition, silica gel was modified by monoamine, ethylenediamine, and diethylenetriamine, and its sorption properties for Co^2+^ and Fe^3+^ in aqueous solution were investigated [37]. However, these studies mainly focus on the preparation of ethylenediamine-silica hybrid materials and the experimental phenomena of adsorbing heavy metal ions under different conditions, while the reaction mechanism of ethylenediamine-silica hybrid materials adsorbing metal ions is rarely reported.

In this work, the ethylenediamine modified silica gel (en/SG) is synthesized and used for the separation of Co(II) and Ni(II). In the IEFPCM solvent model, the structural parameters of the products after the combination of Ni(II) and Co(II) with en/SG are investigated by quantum chemical calculations. Then, the structure of en/SG after sorption of Co(II) and Ni(II) and the mechanism of enhanced separation are discussed.

## 2. Materials and Methods

### 2.1. Synthesis of the Ethylenediamine Modified Silica Gel

Silica gel (SG) is provided by Sinopharm Chemical Reagent Co., Ltd., China. First, 20 g SG and 100 mL HCl (6 mol/L) are added to 200 mL round bottom flask and reacted at 300 K for 4 h. The activated silica gel (ASG) is obtained by filtration, washing, and drying at 400 K for 8 h. SOCl_2_ is added to the activated silica gel and heated at 360 K for 2 h. Then, it is stirred for 10 min, washed with toluene, and dried to obtain the product ASG-Cl. Potassium t-butoxide (t-BuOK) (3 mM) and tetrabutylammonium bromide (n-Bu_4_NBr) (0.5 mM) are added to the cooling solution of N,N-Dimethylformamide (DMF) of 1,2-ethylenediamine and stirred at 0 °C for 15 min. The ASG-Cl (2.0 mM) is added to the above solution and stirred for 24 h. After filtration, washing, and drying, 1,2-ethylenediamine modified silica gel (en/SG) is finally obtained. The schematic diagram of the synthesis process is as follows.

$$\frac{Si-Cl}{THF}+\frac{t-BuOK+n-{Bu}_{4}NBr+C_{2}H_{8}N_{2}}{0{}^{\circ}CTHF}\overset{25{}^{\circ}C24h}{\to }Si-C_{2}H_{8}N_{2}$$

### 2.2. Adsorption Experiment

Configure 0.002 mol/L NiSO_4_·6H_2_O and CoSO_4_·7H_2_O solution, using H_2_SO_4_ and NaOH to adjust the pH value of the solution. Take 250 mg adsorbent into a 150 mL beaker containing 50 mL prepared solution. The adsorption standing time is 40 min. The M^2+^ concentration is measured via ICP-OES (Agilent 5800VDV). The emission wavelength of nickel is 220.141 nm and cobalt is 230.901. The removal efficiency (%) and equilibrium adsorption amount of M(II) (M-Ni, Co) by the adsorbent are calculated according to Equations (1) and (2), respectively, where *η* is the removal efficiency (%) of M (II), C is concentration, q*_e_* is equilibrium adsorption amount of M (II), V is the volume of the adsorbed solution, and m is the mass of the used adsorbent. The separation factor is defined as the ratio of *η_Co_* to *η_Ni_*.

(1) $$\eta =\frac{C_{0}-C_{e}}{C_{0}}\times 100\%$$

(2) $$q_{e}=\frac{(C_{0}-C_{e})V}{m}$$

### 2.3. Structural Characterization

FTIR spectra are collected through Bruker IFS 66 V/S spectrometer in the region of 4000–500 cm^−1^. The morphologies and micro-structures of the prepared samples are characterized by a scanning electron microscope (SEM) (Zeiss Ultra-plus SEM).

### 2.4. Computational Methods

Calculations are done by DFT model in Gaussian 16. The PBE in conjunction by def2SVP basis set is utilized to optimize the structures in this work, which has been widely applied to study complexes [38]. Empirical dispersion corrections from Grimme (D3) are also incorporated during this step [39]. The Ni(II) and Co(II) complexes are optimized and the frequencies of the optimized structures are calculated by the DFT and def2SVP basis sets. Single point energy and ΔG are obtained by the M06-2X and def2-TZVP basis sets. The IEFPCM model is used to calculate the solvent effect [39,40].

## 3. Results and Discussion

### 3.1. Ethylenediamine Modified Silica Gel

**Figure 1:**
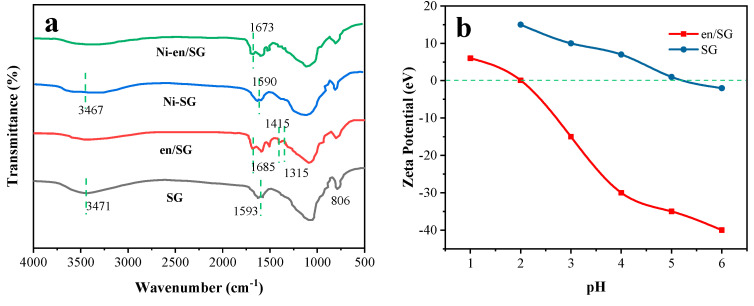
(**a**) FTIR spectra of SG and en/SG before and after adsorption of Ni(II); (**b**) Zeta potential of SG and en/SG as a function of solution pH (adsorbent dosage = 5 g/L, T = 25 °C).

**Figure 2:**
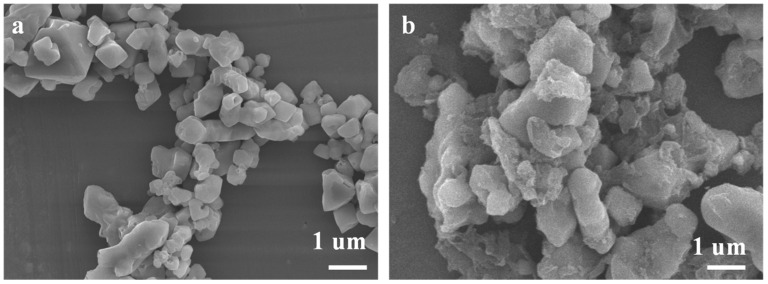
SEM images of (**a**) SG and (**b**) en/SG.

Figure 1 records the FTIR spectra of SG and en/SG in the range of 4000–500 cm^−1^. The FTIR spectrum of SG shows that the band at 806 cm^−1^ is the symmetric stretching of Si-O-Si and that the bands at 3471 and 1593 cm^−1^ correspond to the stretching and bending of O-H bond in silica. In the FTIR spectrum of en/SG, the band at 1685 cm^−1^ is the N-H vibration, and the bands at 1415 and 1315 cm^−1^ correspond to the C-H stretching and bending vibrations, respectively, indicating the successful modification of ethylenediamine to the silica surface. In Figure 2, the SEM results show the morphological characteristics of SG and en/SG obtained at 10 kV. The surface of the silica gel became rough after functionalization, which further illustrated the modification of ethylenediamine. In addition, the acid-base properties and charged state of the surface of the material were analyzed by the Zeta potential. As shown in Figure 1b, the potential of the two materials changes from positive to negative with the increase of pH. This is because the increase of hydroxide in the solution will gradually consume H^+^ on the surface of the material, making it change to a negatively charged state. For en/SG, in a strong acid solution environment, the-NH_2_ on the surface of the aminated silica gel is positively charged, which affects the chelation of the amine group to metal ions. Therefore, the increase of pH value is beneficial to the sorption of metal ions.

### 3.2. Comparison of the Adsorption Properties of Ni and Co on SG and en/SG

**Figure 3:**
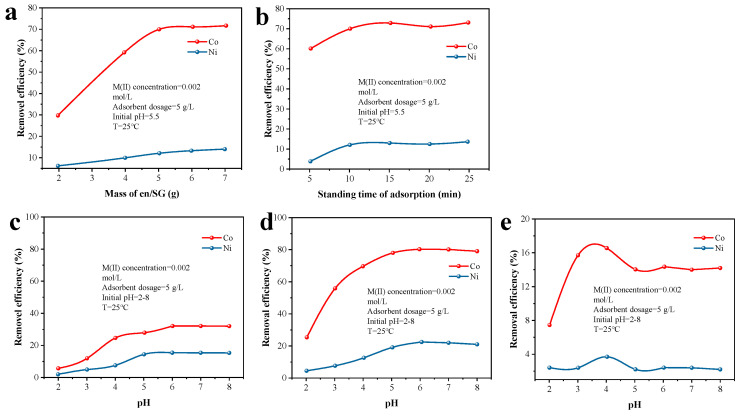
Adsorption and separation efficiency of Co(II) and Ni(II). (**a**) Effect of en/SG dosage on adsorption efficiency. (**b**) Effect of en/SG standing time on adsorption efficiency. (**c**) Effect of pH on SG adsorption efficiency. (**d**) Effect of pH on en/SG adsorption efficiency. (**e**) Effect of pH on separation factor.

A batch process was employed for the sorption of the Ni(II) and Co(II) salt solutions on the surface of SG and en/SG to evaluate its adsorptive characteristics in aqueous media at room temperature. Under normal experimental conditions, the concentration of metal ions in the solution was described according to the experimental steps. The pH of the solution was controlled to 5.5, and the adsorption was static for 40 min, unless otherwise specified. Table 1 and Figure 3 show the results of the sorption of Co(II) and Ni(II) by SG and en/SG under different conditions and the comparison of the separation factors. It can be seen from Figure 3a,b that the reaction has reached equilibrium when the addition amount of en/SG is 5 g and the standing time is 10 min. According to Figure 3c,d, aqueous pH (range 2–6) has an important effect on adsorptive uptake of metal ions. The experimental results show that the removal rate of metal ions increases with increasing pH value in the range of 2–6. A plausible explanation is that the hydrogen ions in the solution have a great influence on the active sites on the surface of the adsorbent. At lower pH values, with the increase of hydrogen ion concentration, the competition with metal ions on the binding site of the adsorbent is stronger, which reduces the adsorption capacity of metal ions. At higher pH values, the presence of hydrogen ions in the solution is reduced, and the surface of the adsorbent is also deprotonated, increasing the adsorption of metal ions. Compared to Figure 3c,d, it can be seen that after modification with ethylenediamine (en/SG), the SG removal efficiency for nickel and cobalt is significantly increased. In addition, the separation factor is defined as the ratio of η_Co_ to η_Ni_. The results show that en/SG has a higher separation performance compared to SG (Figure 3e).

**Figure 4:**
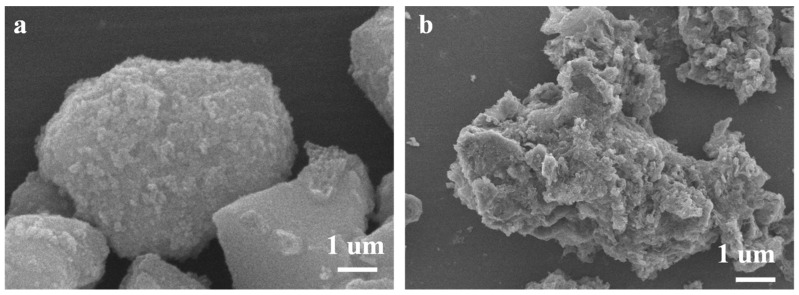
SEM images of (**a**) SG after adsorption of Ni(II), (**b**) en/SG after sorption of Ni(II).

After adsorbing nickel ions, the surface of SG and en/SG became rough, indicating that metal ions were adsorbed by the adsorbent (Figure 4). In addition, the infrared spectra further confirmed the sorption of nickel ions. As shown in Figure 1a, after the sorption of nickel ions, the O-H stretching vibration peak of SG becomes weaker, and the N-H vibration peak of en/SG moves in a negative direction, indicating that the sorption of nickel ions changes the functional group characteristics of the adsorbent surface. The change of N-H characteristic peak position indicates that nickel ions interact with N sites, which will be further elaborated in subsequent text.

**Figure 5:**
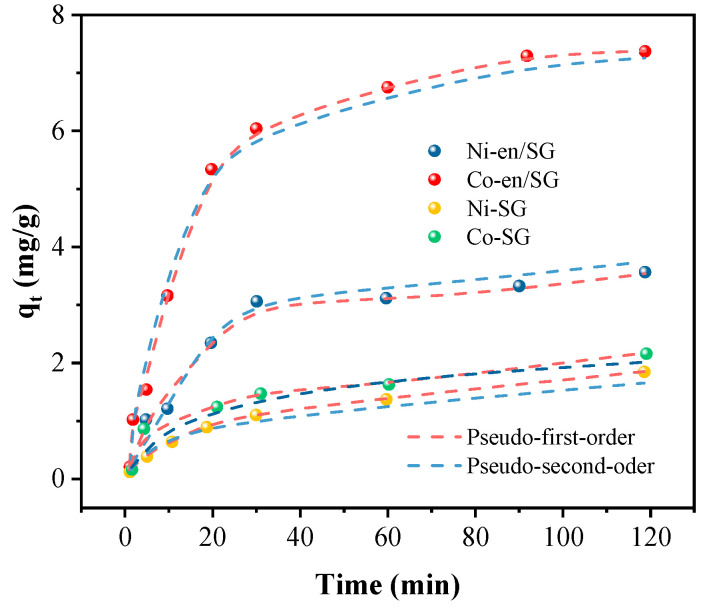
Adsorption kinetics of metal ions on relevant adsorbents (adsorbent dosage = 5 g/L, initial pH = 5.5, T = 25 °C).

The adsorption kinetics can effectively reflect the adsorption rate of metal ions by the reaction material, which can be fitted by pseudo-first-order kinetic and pseudo-second-order kinetic models. The adsorption kinetics of SG and en/SG for nickel and cobalt ions are shown in Figure 5. The kinetic parameters in the adsorption process of metal ions were calculated by two kinetic simulations. The results show that the sorption of nickel and cobalt ions by en/SG is more in line with the pseudo-second-order kinetic model, while the adsorption process of SG conforms to the pseudo-first-order kinetics, and the sorption rate of en/SG is faster than that of SG. Therefore, the sorption of en/SG is a chemical sorption process, while SG adsorption is closer to the physical adsorption process.

**Figure 6:**
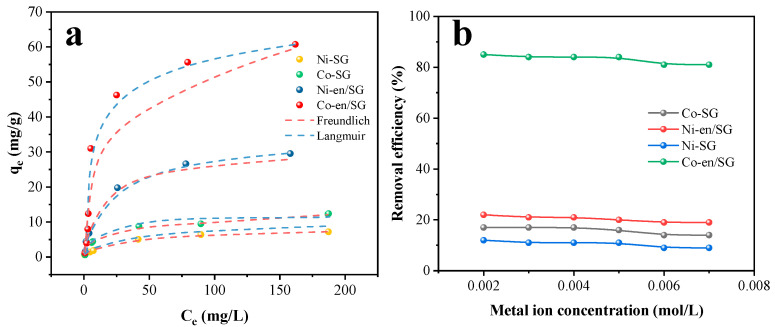
(**a**) Adsorption isotherm of metal ions on SG and en/SG (adsorbent dosage = 5 g/L, initial pH = 5.5, T = 25 °C); (**b**) The influence of initial metal ion concentration to adsorption efficiency (adsorbent dosage = 5 g/L, intial pH = 5.5, T = 25 °C).

**Figure 7:**
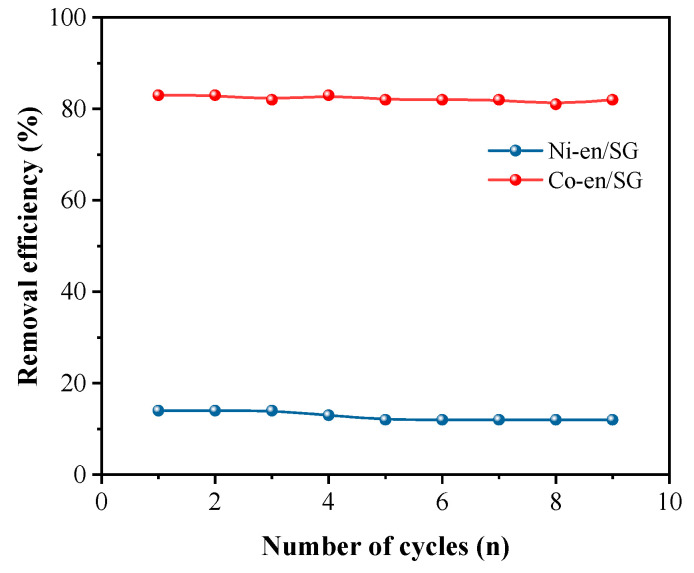
Regeneration sorption of Ni(II) and Co(II) using en/SG for nine cycles of use.

Besides, the adsorption isotherm can effectively reflect the adsorption capacity of the material to metal ions and is fitted by the Langmuir model and Freundlich model. The adsorption isotherms of SG and en/SG for metal ions are shown in Figure 6. Comparing the correlation coefficient (R^2^), the adsorption process of nickel and cobalt on SG material is more in line with the Freundlich model, while the sorption process of en/SG material is more in line with the Langmuir model. Therefore, the sorption of metal ions by en/SG materials is monolayer adsorption, which satisfies the chemical adsorption process, while the adsorption of SG conforms to the multi-layer adsorption process. Generally, the concentration of metal ions in the solution will affect the adsorption efficiency. In order to explore the effect of the initial solution concentration on adsorption performance, the initial solution concentration was changed. The results showed that en/SG could still maintain a good adsorption efficiency with the increase in metal ion concentration (Figure 6b). The regeneration capability of en/SG was evaluated by cyclic sorption tests. The metal ions were eluted from the adsorbent by configuring a certain concentration of hydrochloric acid solution. As depicted in Figure 7, the Co(II) removal efficiency exceeded 80.0% after 9 cycles of reuse, and the removal rate of Ni(II) also remained almost unchanged. It can be inferred that the en/SG showed excellent reusability within an expected lifetime that allowed for its long-term use.

### 3.3. Co(II) and Ni(II) Stable Species in Sulfate Solution

**Figure 8:**
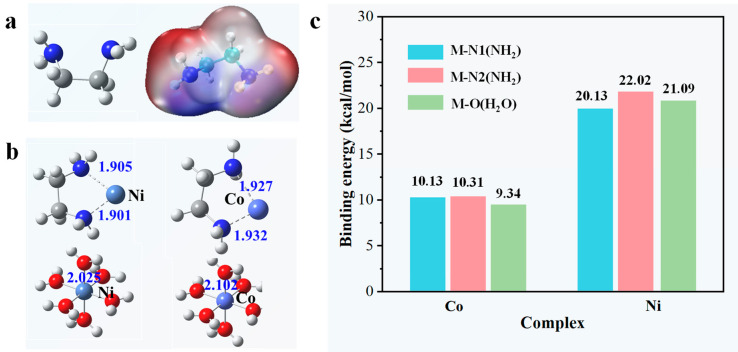
(**a**) Structure of ethylenediamine and its molecular electrostatic potential (ESP) map observed from the vertical direction. (**b**) Bond length between M^2+^ and ethylenediamine. (**c**) The binding energy of M-N and M-O.

The benefits for the en/SG are evident regarding its separation performance, pointing to the grafted ethylenediamine active group. Therefore, the interaction mechanism of ethylenediamine with nickel and cobalt ions are investigated. Figure 8a is the molecular structure of ethylenediamine. It can be seen from the diagram that the two N atoms in the ethylenediamine molecule are locally electron-rich, which will combine with positively charged metal ions. The optimization results of Figure 8b show that the bond length of M-N(NH_2_) is shorter than that of M-O(H_2_O), indicating that metal ions have stronger binding abilities with the NH_2_ group. Therefore, when the en/SG adsorbent is added to the solution, the NH_2_ group competes to replace the water molecules around the metal ions and preferentially form the M-N bond. To further probe the enhanced separation performance, the binding energy between ethylenediamine and metal ions are calculated (Figure 8c). As a result, M-N has a stronger bond energy than M-O, whether it is a nickel ion or cobalt ion. Therefore, ethylenediamine tends to coordinate with metal ions and replace coordinated water molecules to form bidentate ligands. Besides, one ethylenediamine molecule can substitute two coordination water molecules.

**Figure 9:**
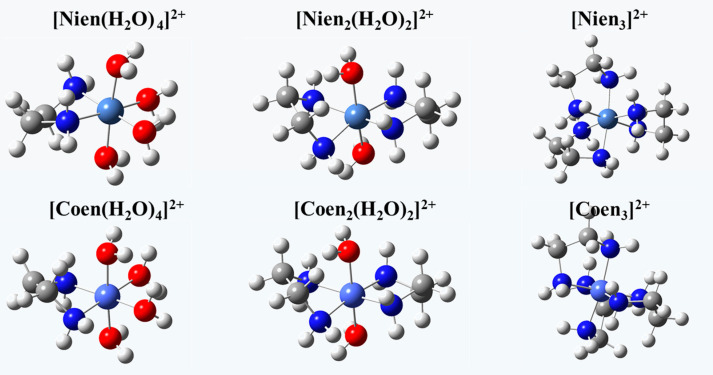
Structure of the species of Ni(II) and Co(II) in aqueous solution after the sorption of en/SG.

As mentioned above, ethylenediamine can replace the coordination water molecules around metal ions, but its coordination number is still unclear. In order to solve this problem, all possible species formed by the coordination of ethylenediamine with metal ions are considered. In the initial solution, nickel and cobalt ions exist in the form of Ni(H_2_O)_6_^2+^ and Co(H_2_O)_6_^2+^ in a sulfuric acid aqueous solution, respectively [41,42,43]. Since ethylenediamine forms a bidentate ligand with metal ions, three species may be formed when ethylenediamine replaces the coordinated water molecules of nickel or cobalt ions, namely [M(en)(H_2_O)_4_]^2+^, [M(en)_2_(H_2_O)_2_]^2+^, and [M(en)_3_]^2+^. The molecular structures of these species are optimized at the B3LYP/6-31G* level (Figure 9).

**Figure 10:**
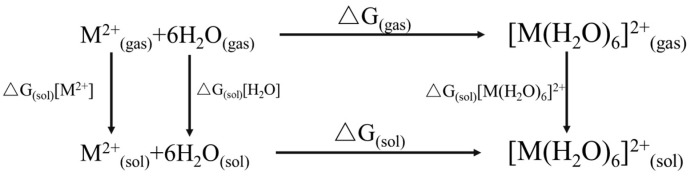
Thermo-dynamic cycle for the hydration of the M^2+^ (M-Ni or Co) [45].

In order to investigate the coordination reactivity of metal ions with ethylenediamine in an aqueous solution, the reaction process is divided into the hydration reaction of metal ions and the complexation reaction of hydrated metal ions with ethylenediamine. In the following calculation, the temperature and pressure of the reaction system are 298 K and 1 atm, respectively. The hydration reaction equation is shown in Equation (3), and the calculation method of the Gibbs free energy is shown in Figure 10, which refers to the calculation method of Hu et al. [43,44,45]. The calculated method for the complexation reaction of hydrated ions with ethylenediamine in line with the hydration reaction and the reaction equation is shown in Equation (4).

(3) $$M^{2+}+6H_{2}O\overset{}{\to }M{\left(H_{2}O\right)}_{6}{}^{2+}$$

(4) $${\left[M{\left(H_{2}O\right)}_{6}\right]}^{2+}+nen\overset{}{\to }{\left[M{\left(en\right)}_{n}(H_{2}O)\right]}_{6-2n}{]}^{2+}+2nH_{2}O$$

The thermodynamic energy changes in the two processes are listed in Table 2. ΔG_sol_ represents the hydration of metal ions, ΔG_n_ represents the complexation of hydrated metal ions with ethylenediamine, and n represents the coordination number of ethylenediamine. It is shown that the absolute values of the Gibbs free energy changes for all three reactions decreased gradually as the number of ethylenediamine molecules substituted for hydrated molecules increased, which may be due to the presence of spatial site resistance in the coordination process. In addition, when the central ion is Co^2+^, the absolute value of the Gibbs free energy change of the reaction system is relatively small due to its Jahn-Teller effect. Accordingly, the reaction is easier and the resulting complexes are more stable. Therefore, after binding with ethylenediamine, both Ni and Co ions exist as [Men(H_2_O)_4_]^2+^ species.

### 3.4. The separation Mechanism of en/SG

**Figure 11:**
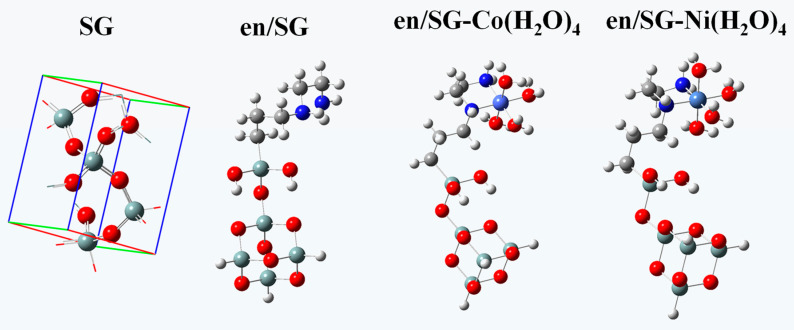
The optimized conformation of SG, en/SG, en/SG-Co(H_2_O)_4_, and en/SG-Ni(H_2_O)_4_.

According to the stable conformation formed by the interaction between ethylenediamine and Ni/Co hydrated ions, the mechanism of the en/SG separation of Ni(II) and Co(II) is further studied. Firstly, the conformations of en/SG are optimized before and after adsorption of Co^2+^ and Ni^2+^ (Figure 11).

By comparing the structural parameters of en/SG before and after metal ions adsorption, the results show that the surface structure of en/SG changes little, and the main change comes from the longer C-N bond in ethylenediamine (Table 3). This is because the interaction between the N atoms in ethylenediamine and the metal ions leads to the weakening of the C-N bond energy and the increase of the bond length. Comparing the bond lengths of Co-N and Ni-N bonds, it can be seen that the two N atoms on the ethylenediamine group have a shorter distance from Co^2+^, indicating that the interaction between ethylenediamine and Co^2+^ is stronger. Besides, the complexation of en/SG with M^2+^ leads to a change in charge density around the metal ions, and the transfer of electrons from the nitrogen atoms on the ethylenediamine to the metal ions. The results show that en/SG transfers 0.45 and 0.27 electrons to Co^2+^ and Ni^2+^, respectively. Therefore, the electron cloud overlap between en/SG and Co^2+^ is larger than that of Ni^2+^ during the adsorption process, and the corresponding coordination bond formed by the former is stronger.

Furthermore, the ΔH and ΔG of the reaction are calculated, and the reaction equation is shown in Equation (5), which corresponds to the substitution of the ethylenediamine group in en/SG for two molecules in the hydrated metal ions. The ΔH and ΔG of the adsorption process are calculated by Equations (6) and (7), respectively. As shown in Table 4, the adsorption enthalpy of [Co(H_2_O)_6_]^2+^ and [Ni(H_2_O)_6_]^2+^ are −122.33 kJ/mol and −90.29 kJ/mol, respectively. The adsorption Gibbs free energies of [Co(H_2_O)_6_]^2+^ and [Ni(H_2_O)_6_]^2+^ are −134.13 kJ/mol and −96.50 kJ/mol, respectively. The ΔG of two metal ions adsorption processes is less than ΔH, indicating that they are exothermic reactions. Comparing the ΔG of the two metal ion adsorption processes, it can be seen that when en/SG adsorbs [Co(H_2_O)_6_]^2+^, the ΔG of the reaction system decreases more, indicating that the reaction is easier to proceed and the formed complex is more stable. Therefore, the en/SG is inclined to adsorb Co^2+^ ions in the mixed solution of nickel and cobalt.

(5) $$en/SiO_{2}+{\left[M{\left(H_{2}O\right)}_{6}\right]}^{2+}{}_{\left(aq\right)}\overset{}{\to }en/SiO_{2}-{\left[M{\left(H_{2}O\right)}_{4}\right]}^{2+}{}_{\left(aq\right)}+2H_{2}O_{\left(aq\right)}$$

(6) $$△H\left(comp\right)=H_{en/SiO_{2}-{\left[M{\left(H_{2}O\right)}_{4}\right]}^{2+}}\left(aq\right)+2H_{H_{2}O}\left(aq\right)-\left\{H_{\left[M{\left(H_{2}O\right)}_{6}{}^{2+}\right](aq)}+H_{en/SiO_{2}(aq)}\right\}$$

(7) $$△G\left(comp\right)=G_{en/SiO_{2}-{\left[M{\left(H_{2}O\right)}_{4}\right]}^{2+}}\left(aq\right)+2G_{H_{2}O}\left(aq\right)-\left\{G_{\left[M{\left(H_{2}O\right)}_{6}{}^{2+}\right](aq)}+G_{en/SiO_{2}(aq)}\right\}$$

Comparing the adsorption process of nickel and cobalt ions by SG and en/SG, the results show that the adsorption mechanism of both changed fundamentally. The adsorption of nickel and cobalt ions by SG is a physical adsorption process, so its separation performance is limited. In comparison, en/SG has strong coordination with metal ions, and the nitrogen in ethylenediamine has different binding ability with nickel and cobalt ions. Therefore, en/SG not only has excellent nickel and cobalt ion sorption performance, but also has good separation characteristics. The above discussion further reveals the mechanism of ethylenediamine modified SG to enhance its separation performance of nickel and cobalt and provides theoretical guidance for improving its performance.

## 4. Conclusions

The separation factor of Ni^2+^ and Co^2+^ increases by 5 times after the surface of silica gel is modified by 1,2-ethylenediamine. The relationship between the active group structure, bond energy, and sorption performance are pointed out by the DFT method. The optimized structure shows that the complexes of Ni(II) and Co(II) have similar coordination modes and exist in the form of [Men(H_2_O)_4_]^2+^ after adsorption. M-N bond analysis shows that the complex formed by Co(II) and ethylenediamine is more stable than Ni(II) in an aqueous solution. In addition, the ΔG of the complex formed by Co(II) and ethylenediamine is more negative than that of Ni(II), therefore ethylenediamine on the surface of adsorbent is inclined to coordinate with Co(II). It can be concluded that the difference of complexing force is the driving force widening the difference between Ni(II) and Co(II) sorption performance on the ethylenediamine modified silica gel. This work provides an insight into the mechanism of amino adsorption of Ni(II) and Co(II), which will help to design adsorbents with higher adsorption properties.

## Figures and Tables

**Table 1:** The adsorption efficiency of SG and en/SG for metal ions (Co(II)\Ni(II)) under different pH conditions.

Adsorbent	Species	pH = 2	pH = 3	pH = 4	pH = 5	pH = 6
SG	Ni	3.1%	5.3%	8.6%	9.5%	11.2%
	Co	4.4%	8.1%	10.4%	13.4%	17.9%
en/SG	Ni	4.5%	6.8%	10.2%	12.2%	14.3%
	Co	5.9%	21.3%	44.8%	61.7%	78.2%

**Table 2:** The Gibbs free energy (ΔG, kcal/mol^−1^) of each reaction processes in the aqueous phase based on B3LYP/6-31G* level.

	M-Co	M-Ni
ΔG_sol_(M^2+^): kJ/mol	−1812.91	−1794.92
ΔG_1_(comp): kJ/mol	−120.96	−97.25
ΔG_2_(comp): kJ/mol	−110.17	−86.69
ΔG_3_(comp): kJ/mol	−45.17	−76.15
ΔG_total_ = ΔG_sol_ + ΔG_1_	−1933.87	−1892.17

**Table 3:** Structure parameters of en/SG, M-en/SG.

en/SG	en/SG Bond Lengths (Å)	en/SG-Ni(H_2_O)_4_ Bond Lengths (Å)	en/SG-Co(H_2_O)_4_ Bond Lengths
R(Si1-O2)	1.654	1.653	1.652
R(Si8-O2)	1.669	1.670	1.669
R(Si1-O2)	1.606	1.607	1.605
R(N24-C27)	1.457	1.535	1.522
R(N32-C26)	1.464	1.532	1.520
R(M-N24)	-	1.441	1.432
R(M-N32)	-	1.440	1.431

**Table 4:** The thermodynamic data of Co^2+^ and Ni^2+^ adsorption on en/SG in aqueous solution.

Thermodynamic Parameters	M-Co	M-Ni
ΔH(comp) (KJ/mol)	−122.33	−90.29
ΔG(comp) (KJ/mol)	−134.13	−96.50

## Data Availability

The authors do not have permission to share data.
